# Hot spots of DNA double-strand breaks in human rDNA units are produced *in vivo*

**DOI:** 10.1038/srep25866

**Published:** 2016-05-10

**Authors:** Nickolai A. Tchurikov, Dmitry V. Yudkin, Maria A. Gorbacheva, Anastasia I. Kulemzina, Irina V. Grischenko, Daria M. Fedoseeva, Dmitri V. Sosin, Yuri V. Kravatsky, Olga V. Kretova

**Affiliations:** 1 Department of Epigenetic Mechanisms of Gene Expression Regulation, Engelhardt Institute of Molecular Biology, Moscow, 119334, Russia; 2 Department of Genomic Diversity and Evolution, Institute of Molecular and Cellular Biology SB RAS, Lavrentiev Ave. 8/2, Novosibirsk, 630090, Russia; 3 Department of Medicine, Novosibirsk State University, Pirogova str. 2, Novosibirsk 630090, Russia; 4 Department of Natural Science, Novosibirsk State University, Pirogova str. 2, Novosibirsk 630090, Russia

## Abstract

Endogenous hot spots of DNA double-strand breaks (DSBs) are tightly linked with transcription patterns and cancer genomics^**1,2**^. There are nine hot spots of DSBs located in human rDNA units^3–6^. Here we describe that the profiles of these hot spots coincide with the profiles of γ-H2AX or H2AX, strongly suggesting a high level of *in vivo* breakage inside rDNA genes. The data were confirmed by microscopic observation of the largest γ-H2AX foci inside nucleoli in interphase chromosomes. In metaphase chromosomes, we observed that only some portion of rDNA clusters possess γ-H2AX foci and that all γ-H2AX foci co-localize with UBF-1 binding sites, which strongly suggests that only active rDNA units possess the hot spots of DSBs. Both γ-H2AX and UBF-1 are epigenetically inherited and thus indicate the rDNA units that were active in the previous cell cycle. These results have implications for diverse fields, including epigenetics and cancer genomics.

DNA breakage occurs during different physiological events in nuclei, including replication, transcription, and genome rearrangements. Exogenous factors, such as chemicals, radiation, viral infection, and a high-salt environment, also challenge the DNA integrity. Massive DNA fragmentation has been described in cancer cells and during the early course of a neurodegenerative disease^1^,^2^. It cannot be excluded that there are physiological cellular mechanisms that result in limited or extensive DNA breakage followed by repair in diverse cell types at different stages of development in which distinct enzymes may operate. There would be dramatic consequences for genomic integrity should these mechanisms go awry in response to different endogenous or environmental challenges. Recently, a technique for genome-wide mapping of double-strand breaks (DSBs) with one-nucleotide precision was developed that led to the discovery of hot spots of DSBs that delimit coordinately expressed domains in Drosophila and human cells^3^,^4^. Nine hot spots of DSBs, denoted Pleiades, were detected by this technique in human rDNA genes inside the intergenic spacer (IGS)^5^,^6^. The profile of these hot spots detected in HEK293T cells coincides with the profiles of CTCF binding sites and H3K4me3 marks in rDNA units from twelve more human cell lines. The data strongly suggest the conserved organization of some unknown structural/functional elements in the IGS and a strong link between Pleiades and transcription inside rDNA clusters^6^. In order to investigate the nature of hot spots of DSBs detected in the most actively transcribed genes in the human genome, i.e., rDNA genes, and especially whether these hot spots are generated *in vivo* or *in vitro* during the procedure used for genome-wide mapping of DSBs, we performed the current study. Here, we report that the hot spots are generated *in vivo* only inside transcriptionally active rDNA clusters. Together with the data that rDNA units are among the most fragile sites in the human genome^5^, our data indicate a tight link between active transcription and *in vivo* DNA breakage. We also propose a model to explain the possible origin and the properties of hot spots of DSBs inside rDNA units.

## Results

**Figure 1:**
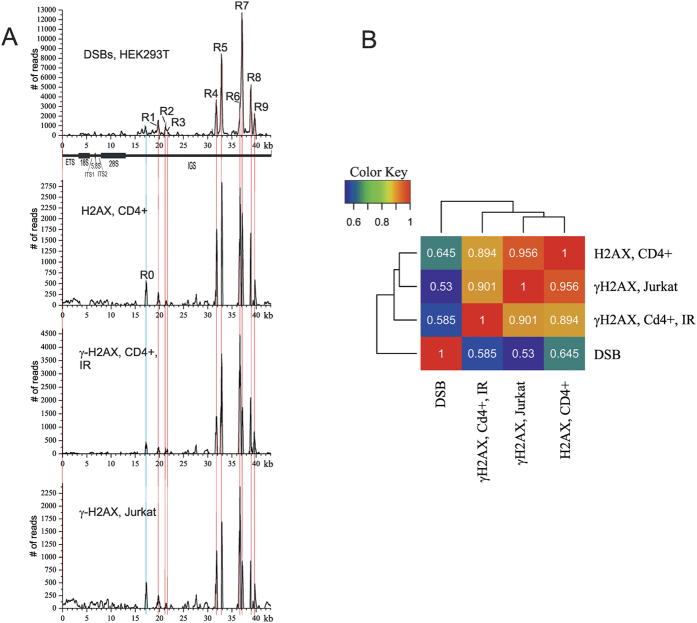
The relationships between hot spots of DSBs, H2AX, and γ-H2AX marks inside rDNA units. (**A**) The profiles of DSBs, H2AX, and γ-H2AX marks along a rDNA unit are shown. The ChIP-Seq data for CD4^+^ resting cells (irradiated (IR) or not irradiated) and actively proliferating Jurkat cells were used (8). Red lines show the position of nine hot spots of DSBs (R1–R9) inside the IGS. (**B**) Correlation heatmaps of pairwise comparisons between median signals for DSBs in HEK293T cells, and H2AX and γ-H2AX marks in CD4^+^ or Jurkat cells inside the IGS region are shown. Pearson’s correlation coefficient, r, is between 0.53 and 0.64, indicating a strong positive relationship between DSBs, H2AX, and γ-H2AX marks. The thin blue line shows R0 (Region 0), which was visualized by both and H2AX and γ-H2AX marks. The overview of reads representing DSBs in R0 is shown in Fig. S1.

When phosphorylated on serine 139, H2AX is called γ-H2AX and is used as a biomarker of *in vivo* DSBs^7^. Recently, it was shown that H2AX deposition can be an even better indicator of endogenous DSBs than γ-H2AX, which is less specific for endogenous DSBs^8^, which is why we used the available ChIP-Seq data on H2AX marks in human cells for mapping inside 43-kb rDNA units. We observed that the profile of DSBs and profiles of both H2AX and γ-H2AX in CD4^+^ T lymphocytes practically coincide (Fig. 1A). This conclusion was based on a visual inspection of the data regarding correspondence between DSBs and H2AX, and the γ-H2AX marks were also confirmed by calculated correlations. CD4^+^ T lymphocytes are resting cells, and thus are not prone to replication stress^9^. It follows that the observed pattern of H2AX and γ-H2AX should be associated only with active transcription inside rDNA genes. Surprisingly, the same pattern of γ-H2AX that coincided with the profile of DSBs was observed in actively proliferating Jurkat cells (T-cell lymphoma culture cells). This effect could be produced by both replication- and transcription-mediated stress in the cancer cell. These ChIP-Seq data cannot discriminate the inputs coming from replication and transcription in Jurkat cells. Nevertheless, the results shown in Fig. 1 indicate that active transcription is sufficient for such distribution of both H2AX and γ-H2AX marks. In any case, these data strongly suggest that the Pleiades are the result of *in vivo* DNA breakage. These nine regions of IGS are very often subjected to *in vivo* cleavage at specific sites; although the mechanism involved is unknown, we now know that these fragile sites are marked by both H2AX and γ-H2AX marks.

**Figure 2:**
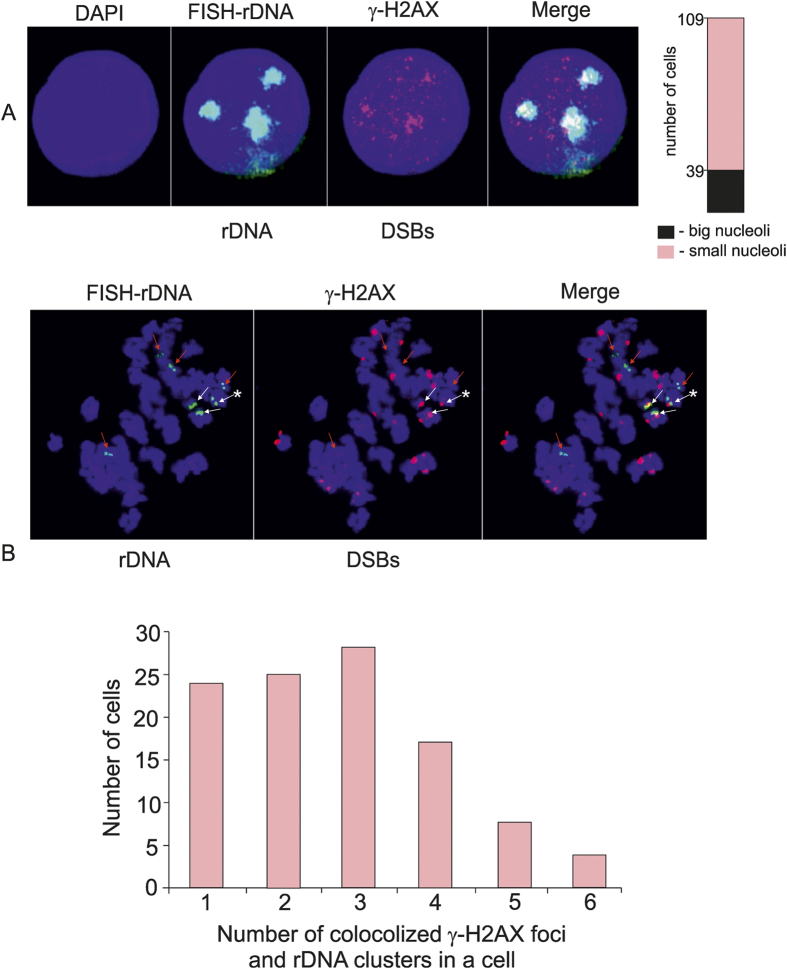
The major γ-H2AX foci are located inside nucleoli. (**A**) The GM06895 lymphoblastoid cell line in interphase was subjected to combined immunostaining and FISH to show the localization of rDNA and γ-H2AX marks. rDNA was visualized by hybridization to a bio-labeled probe containing ETS and a part of the18S gene, and then by FITC-avidin (green). γ-H2AX marks were visualized with antibodies coupled to Alexa-555 (red). The results of population analysis of nucleoli variability in 109 cells are shown by the bar. (**B**) The same cell line in metaphase. Red and white arrows indicate double green dots corresponding to rDNA clusters in homologous chromosomes. White arrows indicate rDNA clusters possessing DSBs visualized by γ-H2AX marks. The white arrow with an asterisk indicates an example in which only one of two homologous chromosomes possesses a γ-H2AX focus. The results of population analysis of numbers of colocalized rDNA clusters and γ-H2AX marks in 105 cells in metaphase are shown by the bars. The number of colocalized sites in a cell vary between 1 and 6.

Next, we decided to check whether this rDNA breakage could be microscopically observed in interphase cells. Figure 2A shows the results of combined immunostaining and fluorescent *in situ* hybridization (immuno-FISH) using anti-γ-H2AX antibodies and a fluorescently labeled rDNA probe. We observed that the largest γ-H2AX foci are located mainly inside central areas of the nucleoli. The rest of the γ-H2AX foci are scattered inside the nucleus. The data obtained in interphase chromosomes from two different cell lines are shown in Fig. S2. The signal from FISH is larger than the co-localized signal from the γ-H2AX staining, and therefore, we suppose that only some part of the rDNA clusters inside each nucleolus, mainly located in the central part of the nucleoli, is labeled by this histone mark. It might follow that not all copies of rDNA genes are subjected to breaks. In any case, the data clearly support the conclusion that rDNA genes are the most fragile sites in human chromosomes^5^, and furthermore, independently confirm the data on the profiles of H2AX in rDNA units that indicate the *in vivo* breakage inside IGS regions (Fig. 1).

We observed that in both GM06891 and GM06895 lymphoblastoid cell lines there is variability in size and number of nucleoli in interphase chromosomes (see Fig. S3). The population analysis revealed that 36% of GM06895 cells possessed big nucleoli (Fig. 1A).

In order to investigate whether γ-H2AX foci are preserved (epigenetically inherited) in only the portion of rDNA clusters that possessed breaks in the previous cell cycle, we analyzed the metaphase chromosomes for individual rDNA clusters. Using combined immuno-FISH, we detected that, in the tested cell line, among seven rDNA clusters—which are visualized as double green dots, each one corresponding to one of the homologous chromosomes—there were only three clusters that co-localized with γ-H2AX foci (Fig. 2B). Interestingly, there is an example demonstrating that only one of the two homologous chromosomes could possess a γ-H2AX focus (Fig. 2B). The data might indicate that only part of the rDNA clusters were active during interphase in the previous cell cycle, possessed transcriptional hot spots of DSBs and γ-H2AX marks, and were epigenetically inherited in metaphase chromosomes. These data also might indicate that γ-H2AX binding sites mark not only rDNA clusters that were active in the previous cell cycle, but also display clusters that will be active during the next cell cycle. The data shown in Fig. 2B demonstrate that, in total, there were about twenty sites that possessed γ-H2AX marks in the metaphase chromosomes. Only three of them corresponded to rDNA units. It follows, therefore, that there are about seventeen genomic sites that also inherited γ-H2AX marks in metaphase. We suppose that these sites also possessed hot spots of DSBs in the previous cell cycle. The population analysis revealed that the number of colocalized γ-H2AX foci and rDNA clusters in GM06895 cells vary between 1 and 6 (Fig. 2B).

**Figure 3:**
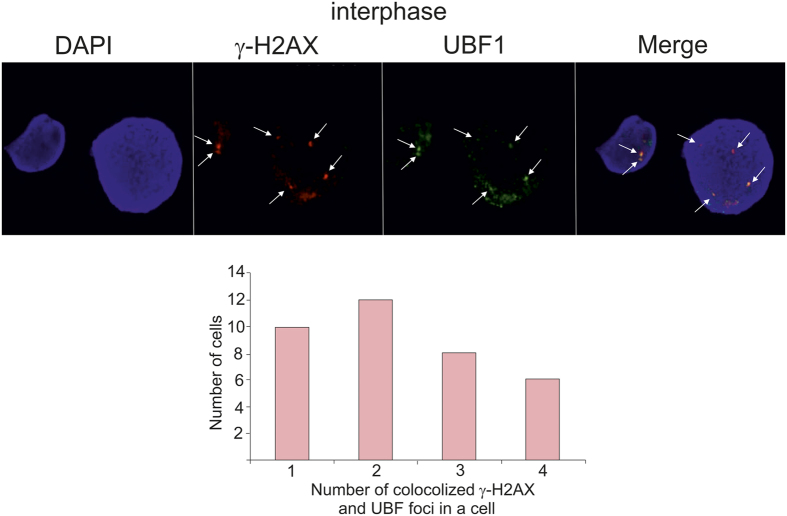
The major γ-H2AX foci and UBF-1 binding sites are co-localized in interphase nuclei. The GM06895 lymphoblastoid cell line in interphase was subjected to immunostaining. γ-H2AX marks were visualized with antibodies coupled to Alexa-555 (red). UBF1 binding sites were visualized with antibodies coupled to FITC (green). Arrows indicate the major spots where the co-localization was observed. The results of population analysis of numbers of colocalized γ-H2AX marks and UBF1 binding sites in 36 cells in interphase are shown by the bars. The number of colocalized sites in a cell vary between 1 and 4.

To test the supposition that only transcriptionally active rDNA clusters possess γ-H2AX marks, we determined whether the major γ-H2AX foci are co-localized with UBF-1 (Pol I-specific upstream binding factor) binding sites. It is known that UBF-1 plays an epigenetic role in the formation and maintenance of active rRNA gene chromatin^10^. Only a fraction of rDNA copies in human cells are transcriptionally active, and UBF-1 marks these active rDNA units^11^,^12^. Figure 3 shows that in interphase chromosomes the main γ-H2AX foci coincide with UBF-1 binding sites. These data clearly support our supposition and indicate that the hot spots of DSBs are present only in active rDNA clusters. The population analysis revealed that γ-H2AX foci and the brightest UBF binding sites in GM06895 cells vary between 1 and 4. In two interphase nuclei, shown in Fig. 3, we observed two and four colocalized sites.

We also observed that γ-H2AX foci in metaphase chromosomes coincide with the binding sites of UBF-1 (Fig. S4). At the same time, we observed numerous scattered small spots corresponding to binding sites of UBF-1 in different metaphase chromosomes. ChIP-Seq of UBF-1 revealed a substantial enrichment in the promoter and coding region of rDNA units, as well as binding to sites throughout the genome that were mainly within 2 kb of transcription start sites (TSS)^13^. We conclude that the observed small spots of UBF-1 binding correspond to these genomic regions.

**Figure 4:**
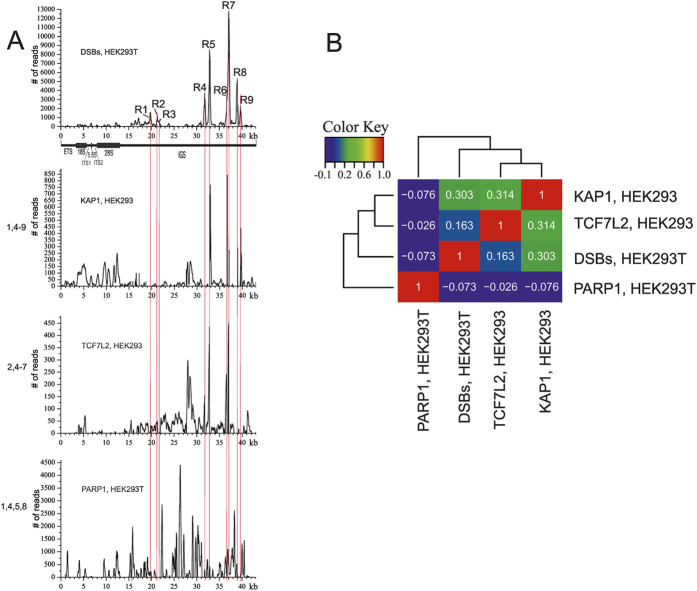
The relationships between hot spot of DSBs, and KAP1, TCF7L2 and PARP1 binding sites inside rDNA units. (**A**) The profiles along the rDNA unit are shown. The thin red lines show the position of nine hot spots of DSBs (R1–R9) inside the IGS. (**B**) Correlation heatmaps of pairwise comparisons between median signals for DSBs in HEK293T cells, KAP1, TCF7L2 (both in HAK293 cells), and PARP1 in HEK293T cells inside the IGS region are shown. The Pearson’s correlation coefficient, r, indicates a moderate positive correlation between DSBs and KAP1 binding sites inside IGS. KAP1 binds within R4 and R6–R9 (similar to T-bet, see Fig. S5 and Table S1), TCF7L2 binds within R2 and R4–R7, and PARP1 slightly binds within R1, R4, R5, and R8.

To answer the question about the nature of Pleiades, which are present only in active rDNA clusters, we analyzed the binding profiles of three master regulators of gene expression in human cells. Our previous data indicate that the binding sites of CTCF precisely coincide with these hot spots of DSBs, suggesting a role for these nine regions of IGS in the regulation of expression of rDNA clusters^5^. In fact, CTCF plays many roles in human cells and can act as transcriptional activator or repressor, or as an insulator, and is involved in the formation of 3D chromatin structures^14^,^15^. We analyzed the profiles of KAP1, TCF7L2, and PARP1 inside the rDNA unit. It is known that the evolutionarily conserved protein KAP1 (KRAB-associated protein 1) regulates the dynamic organization of the chromatin structure by changing epigenetic patterns, chromatin compaction, and DNA repair^16^. KAP1 is an interaction partner of the KRAB (Krüppel-associated box) domain-containing zinc finger transcription factors. We observed that the profiles of KAP1 binding exactly corresponded with seven hot spots of DSBs in rDNA (R1, R4–R9; Fig. 4A). It is not clear whether this result reflects the role of KAP1 in DNA repair, epigenetic regulation, or both. KAP1 is involved in silencing of euchromatic and pericentric heterochromatic regions and is highly enriched at the periphery of nucleoli, suggesting that inactive rDNA clusters are located at the periphery of each nucleolus^17^. On the other hand, the data shown in Fig. 2A and in Fig. S2 suggest that active rDNA clusters possessing Pleiades are located in the central part of each nucleolus and possess γ-H2AX marks, which is why we conclude that KAP1 marks inactive rDNA clusters, while γ-H2AX marks the active ones. KAP1 does not possess a DNA-binding domain and can interact with the chromoshadow domain of HP1 family members^18^. This conclusion is in good agreement with the recent observation that actively transcribed rDNA repeats are positioned within the interior of the nucleolus, and the inhibition of transcription of rDNA clusters by RNA polymerase I is coupled to the movement of rDNA from the nucleolar interior to the periphery, where they are anchored in perinucleolar heterochromatin^19^,^20^. The binding profile of KAP1 (Fig. 4) strongly suggests an important role of IGS regions possessing Pleiades not only in active transcription of rDNA units, but also in its repression. The mechanisms that may operate in both cases are not yet known, but it is clear that the regions where Pleiades are located play a central role in this regulation.

We also found that the binding sites of the TCF7L2 (Transcription factor 7-like 2) master regulator overlaps with five hot spots of DSBs (R2, R4–R7; Fig. 4A). TCF7L2 is involved in activation of various target genes that are switched on upon activation of the Wnt signaling pathway, and plays an important role in regulating cell fate and differentiation during embryogenesis^21^. It was shown recently that many genomic regions that are marked by both H3K4me1 and H3K27Ac also bind TCF7L2, suggesting that TCF7L2 plays an important role in enhancer activity. TCF7L2 co-localizes with a number of transcription factors, including GATA3, which tethers TCF7L2 to some genomic sites^22^. To test whether it might be true and for sites of Pleiades, we determined the GATA3 profile along the rDNA unit. Nevertheless, we observed that GATA3 mainly binds in the coding region, although overlap with the R9 hot spot was also observed (Fig. S5). At the same time, we did not detect any correlation between the main PARP1 binding sites and hot spots of DSBs in rDNA units, although small peaks were observed at R1, R4, R5, and R8 (Fig. 4A), while the major binding sites of PARP1 were observed at the region located between R3 and R4.

## Discussion

It is known that another master regulator, T-bet (T-box transcription factor), shares a large proportion of target genes with GATA3 and was found to be located in nucleoli^23^. We analyzed the profile of T-bet inside rDNA, and observed that it was very similar to that of GATA3 in the coding region (Fig. S5). However, T-bet binding sites inside the IGS overlap with five hot spot of DSBs (R4, R6–R9), indicating its involvement in rDNA regulation.

The sizes and distribution of peaks shown for DSBs and γ-H2AX marks practically coincide, which suggests *in vivo* breakage of rDNA units. At the same time, the binding sites of KAP1, TCF7L2, GATA3, and T-bet transcription factors coincide with different sets of particular hot spots of DSBs inside rDNA units. Previously, these master regulators were considered as important regulators only in specialized cell types. Our data on their binding inside rDNA expand the functional role of these factors. Table S1 shows that several master regulators often bind at a corresponding hot spot. These data suggest that Pleiades correspond to functionally important regions of human rDNA units that are characterized by binding of several master regulators involved in different mechanisms of epigenetic regulation.

Our studies reveal new insights into the organization of human ribosomal genes, and suggest that hot spots of DSBs are produced *in vivo* only in active rDNA clusters and play an important role in epigenetic regulation of these genes that produce about 80% of cellular RNA. The question arises regarding the origin and apparent role of these chromosomal breaks. Our data suggest that Pleiades are not the result of replication-mediated stress, because they occur in the resting CD4^+^ cells, which are not replicating, and that is why we suppose that these hot spots are connected with transcription. At present, however, we cannot determine what comes first: the transcription or the breakage? Whether R-loops can trigger genomic instability and are a source of the DSBs that occur at numerous transcribed regions has been discussed for many years^24^,^25^. There are some data against the supposition of DSBs inside rDNA units. First, Pleaides are located inside IGS, which are not transcribed. Recently, very weak transcription was detected inside IGS, and several R-loop regions were described, which could be a rare transcriptional by-product^13^,^26^. We found that these R-loop regions are located in the regions that do not overlap with the described hot spots of DSBs (Fig. S6), which is why, at present, we cannot consider R-loops as a possible cause of these DSBs. An alternative explanation that we cannot currently exclude is that these breaks are produced *in vivo* in order to quickly load some preexisting multimeric protein complexes at particular regulatory regions inside the IGS (Fig. S7). The studies into the nature and origin of hot spots of DSBs inside rDNA units are important for both the understanding of regulatory mechanisms operating on the most actively transcribed genes in the human genome and for investigations into cancer genomics because in 54% of solid tumors, there are rDNA cluster alterations before the start of the clonal tumor expansion^27^. We believe that our current experiments can address some questions raised by this study.

## Methods

### Computer treatments

Profiles of DSBs, H2A, H2AX, KAP1, TCF7L2, PARP1, and GATA3 along rDNA units were created from raw reads as described previously^13^. Data sets were aligned to rDNA sequences with bowtie v.1, allowing two mismatches per read. Prior to alignment, non-unique reads were removed from each FASTQ file with fastx_collapse for single-end reads and fastuniq^28^ for paired-end reads. During alignment, reads with more than one reportable alignment were discarded using the ‘-m 1’ option. Alignments were converted to BED files by the BamToBed program of the BEDTools package^29^. Profiling was made with F-seq, and the fragment size was set to 200 bp. For all ChIP-Seq data analyzed, the corresponding input samples were analyzed as described above and subtracted at each base from the corresponding base of the ChIP-Seq data using Perl scripts. The resulting filtered profiles were median-smoothed in 100-bp windows, and then Pearson correlation matrices were calculated by Perl scripts. Correlation heatmaps and clusters were created from correlation matrices by the heatmap.2 function from the gplots package of R. The following ChIP-Seq data were used: wgEncodeEH002022 (for TCF7L2 in HEK293 cells); wgEncodeEH001779 (for KAP1 in HEK293 cells); GSE25577 (for H2AX and γ-H2AX in CD4^+^ and Jurkat cells); GSM776558 (for GATA3 in Th1 cells); GSM776557 (for T-bet in Th1 cells); GSE74954 (for PARP1 in HEK293T cells). For profiling of DSBs in HEK293T cells, the data with accession number GSE49302 were used.

### Combined immunostaining and fluorescent *in situ* hybridization (immuno-FISH)

#### Preparation of chromosome spreads

Immortalized human B-lymphocytes, and Epstein Barr virus-transformed GM06895 and GM06891 cell lines were obtained from the Coriell collection (Camden, NJ, USA). Cells were grown in RPMI medium (Life Technologies, Grand Island, NY, USA) with 10% heat-inactivated fetal bovine serum, plus penicillin and kanamycin. Before preparation of spreads, cells were incubated with Colcemid (Gibco) at 100 ng/mL for 8 h. Then the cells were suspended in 1 mL of 0.075 М KCl and incubated for 20 min at 37°С. The spreads were prepared using a Shandon Cytospin 4 (Thermo Scientific) centrifuge. For each slide, about 10 × 10^3^ cells were used. The centrifugation was performed for 5 min at 2000 rpm.

#### Combined immuno-FISH

Immunostaining was performed using primary mouse monoclonal antibodies to γ-H2AX (Abcam, ab18311-100) in KCM buffer (120 mM KCl, 20 mM NaCl, 10 mM Tris/HCl pH 8.0, 0.5 mM EDTA, 0.1% Triton X-100) containing 3% BSA. Incubation was performed for 1 h at room temperature. Incubation with secondary antibodies (Alexa 555-conugated rabbit anti-mouse immunoglobulin, Abcam, 150126) was performed under the same conditions. The spreads were fixed in 4% (v/v) formaldehyde in KCM buffer, and mounted in DAPI/DABCO. The slides were analyzed using a fluorescent Olympus BX53 microscope. Plasmid pHr13, containing ~10 kb of the human rDNA region^30^, was used after labeling with bio-11-dUTP by nick-translation (Nick Translation System, Invitrogen). After immunostaining, the slides were washed with a solution containing 4xSSC and 0.1%Triton X-100 (two times, 5 min each). Then, Proteinase K (Invitrogen) treatment was performed (25 ng/mL in TBS buffer containing 25 mM Tris-HCl pH 7.4, 150 mM NaCl) at room temperature, followed by washing in TBS (two times, 5 min each). For dehydration, consecutive washes with 70%, 80%, and 96% ethanol were performed. The DNA was denatured in 70% formamide in 2xSSC for 4 min at 72°С. The probe was prepared from an ethanol-precipitated mixture containing 0.2 μg of bio-labeled rDNA and 10 μg Cot20 human DNA. The precipitate was dissolved in 20 μL of a solution containing 50% formamide, 10% dextran sulfate, and 2xSSC. The probe was denatured by incubation at 96°С for 8 min. CISS (chromosome *in situ* suppression) was performed by hybridization of a probe for 1 h at 42°С in a wet chamber. Consecutive washes were performed at 42°С in a solution containing 50% formamide and 2xSSC (three times for 3 min each), followed by washing with 2xSSC (two times for 3 min each). Finally, the slides were washed in 0.2xSSC for 3 min. Fluorescein avidin DCS (Vector Laboratories) and biotinylated anti-avidin antibody (Vector Laboratories, BA-0300) were used for staining. The slides were mounted in DAPI/DABCO. The analysis was performed with a fluorescent Olympus BX53 microscope using the coordinates selected after immunostaining.

### Immunostaining

The slides were prepared as described above. Immunostaining was performed using primary rabbit monoclonal antibodies to UBF1 (Abcam, ab75781) and primary mouse monoclonal antibodies to γ-H2AX (Abcam, ab18311-100) as described above. The staining was performed by incubation with secondary antibodies FITC-conjugated goat anti-rabbit immunoglobulin (Sigma, F0382, for visualization of UBF1) and Alexa 555-conugated rabbit anti-mouse immunoglobulin, Abcam, 150126, for visualization of γ-H2AX foci).

## Additional Information

**How to cite this article**: Tchurikov, N. A. *et al.* Hot spots of DNA double-strand breaks in human rDNA units are produced *in vivo*. *Sci. Rep.* **6**, 25866; doi: 10.1038/srep25866 (2016).

## Supplementary Material

Supplementary Information
