# Supplementary material for: Hot spots of DNA double-strand breaks in human rDNA units are produced in vivo
Source: Sci Rep. 2016 May 10;6:25866. doi: 10.1038/srep25866 (PMC4861929; doi:10.1038/srep25866)
Supplement: Supplementary Information [file srep25866-s1.pdf]

Supplemental Information for:

Hot spots of DNA double-strand breaks in human rDNA units are produced *in vivo*

Nickolai A. Tchurikov,[1]† [*] Dmitry V. Yudkin,[2,3]† Maria A. Gorbacheva,[1] Anastasia I. Kulemzina,[2] Irina V. Grischenko,[4] Daria M. Fedoseeva,[1] Dmitri V. Sosin,[1] Yuri V. Kravatsky[1] & Olga V. Kretova[1]

[1] Department of Epigenetic Mechanisms of Gene Expression Regulation, Engelhardt Institute of Molecular Biology, Moscow, 119334, Russia

[2] Department of Genomic Diversity and Evolution, Institute of Molecular and Cellular Biology SB RAS, Lavrentiev Ave. 8/2, Novosibirsk, 630090, Russia

[3] Department of Medicine, Novosibirsk State University, Pirogova str. 2, Novosibirsk 630090, Russia

[4] Department of Natural Science, Novosibirsk State University, Pirogova str. 2, Novosibirsk 630090, Russia

Figures S1–S7

Table S1

[*] Corresponding author. Email: tchurikov@eimb.ru

†These authors contributed equally to this work.

Figures S1–S7:

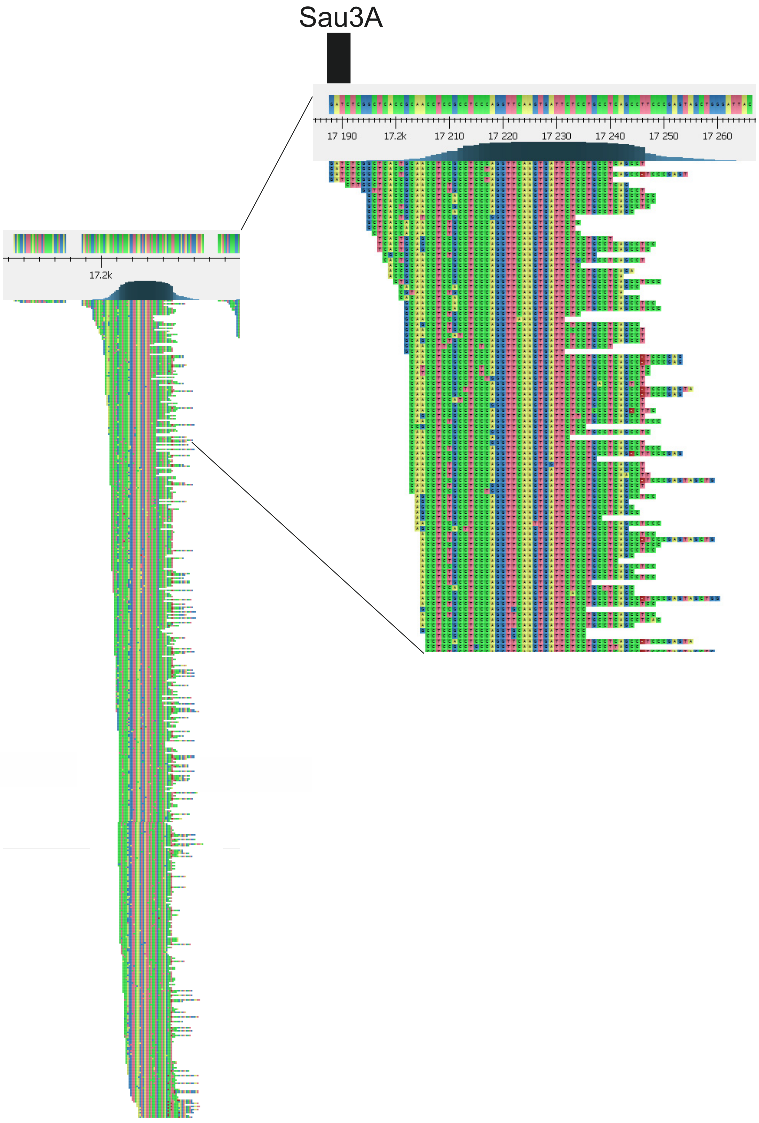

Fig. S1. **Overview of reads mapped in R0.** The corresponding reads representing DSBs are delimited by the cut sites and the Sau3A site, and are schematically visualized using UGENE software (http://ugene.unipro.ru/). The numbering is according to the human ribosomal DNA complete repeating unit sequence (accession number: U13369). The reads in R0 are aligned in 533 rows.

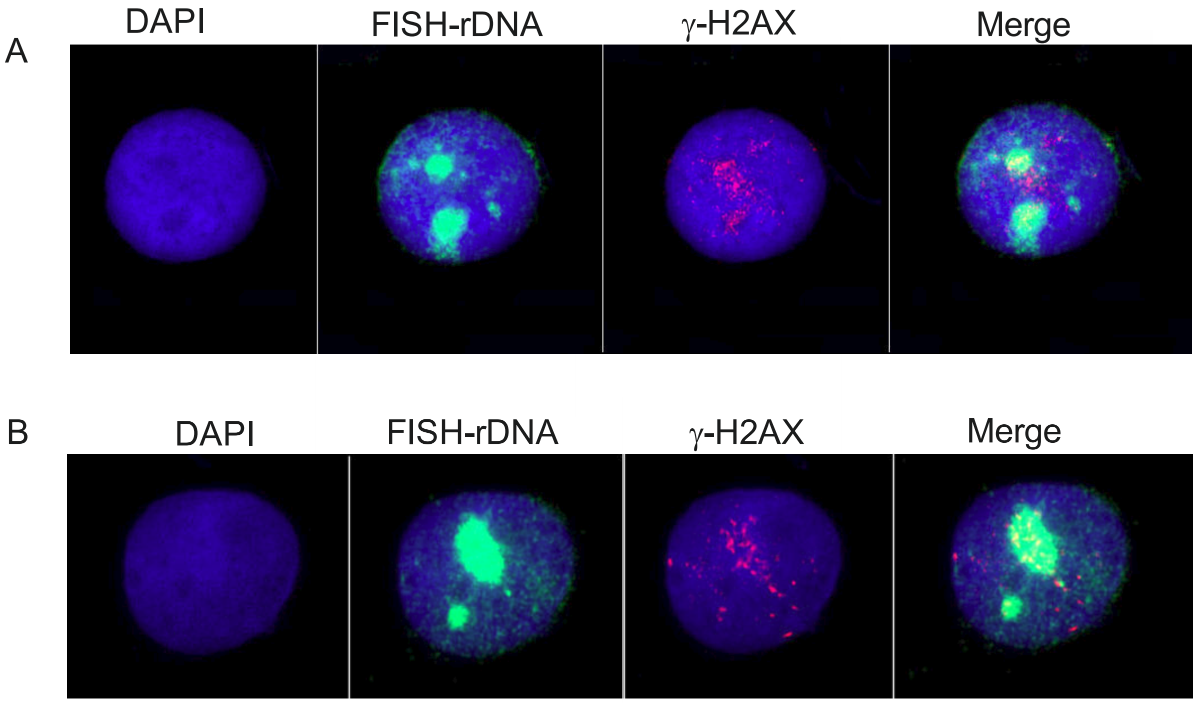

Fig. S2. **The major γ-H2AX foci are located inside nucleoli**. (**A**) Nucleus from the GM06891 lymphoblastoid cell line in interphase that was subjected to combined immunostaining and FISH to show the localization of rDNA and γ-H2AX marks. rDNA was visualized by hybridization to a bio-labeled probe containing ETS and a part of the18S gene, and then by FITC-avidin (green). γ-H2AX marks were visualized with antibodies coupled to Alexa-555 (red). (**B**) Nucleus from the GM06895 lymphoblastoid cell line in interphase that was subjected to the same combined immunostaining and FISH.

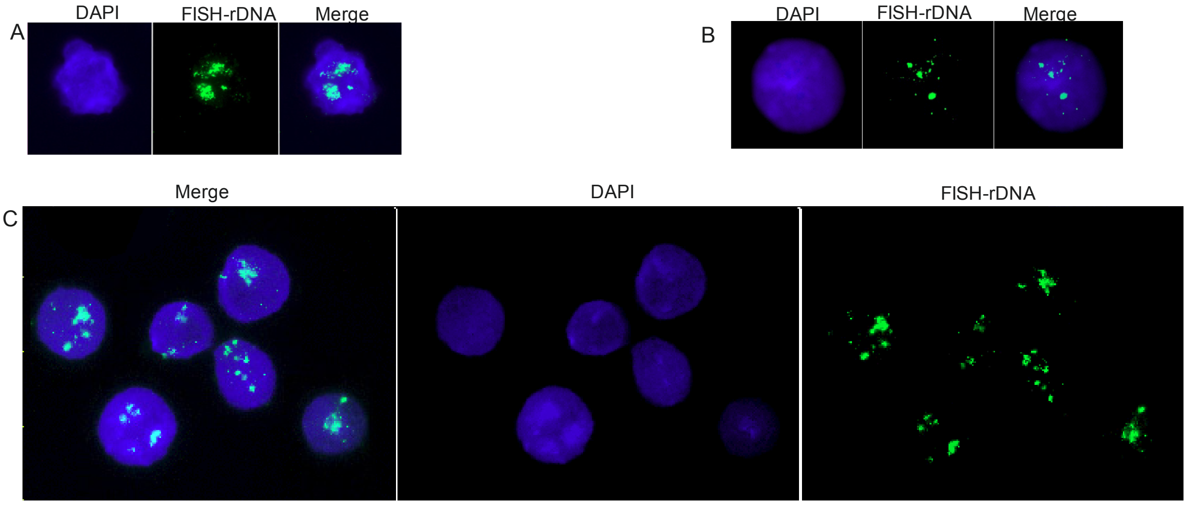

Fig. S3. **Variability of nucleoli in GM06895 lymphoblastoid cell line in interphase**. (**A**) Big nucleoli are visualizes by FISH. (**B**) Small nucleoli. (**C**) Group of six cells represents both small and big nucleoli. Nucleoli were visualized by hybridization to a bio-labeled probe containing ETS and a part of the18S gene, and then by FITC-avidin (green).

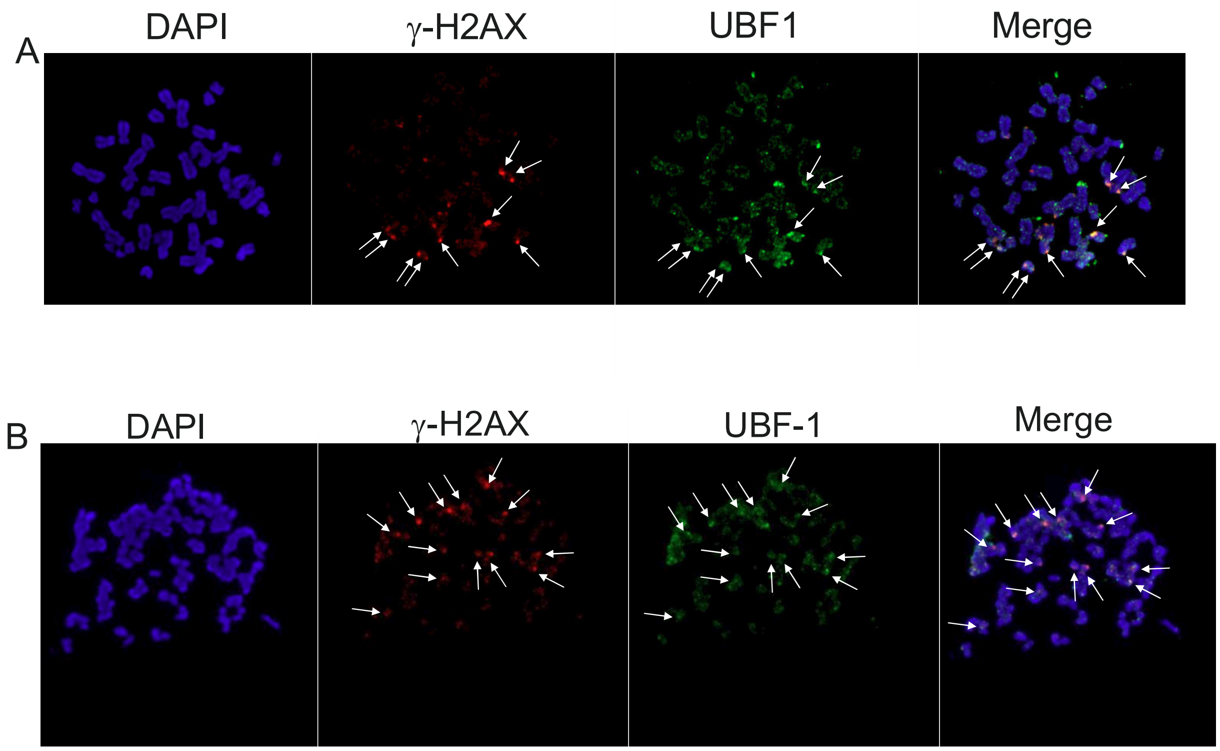

Fig. S4. **The major γ-H2AX foci are located inside nucleoli**. (**A**) and (**B**) show two nuclei from the GM06895 lymphoblastoid cell line in metaphase that were subjected to immunostaining to show localization of γ-H2AX marks and the UBF1 binding sites. γ-H2AX marks were visualized with antibodies coupled to Alexa-555 (red). UBF1 binding sites were visualized with antibodies coupled to FITC (green). Arrows indicate the major spots where co-localization was observed.

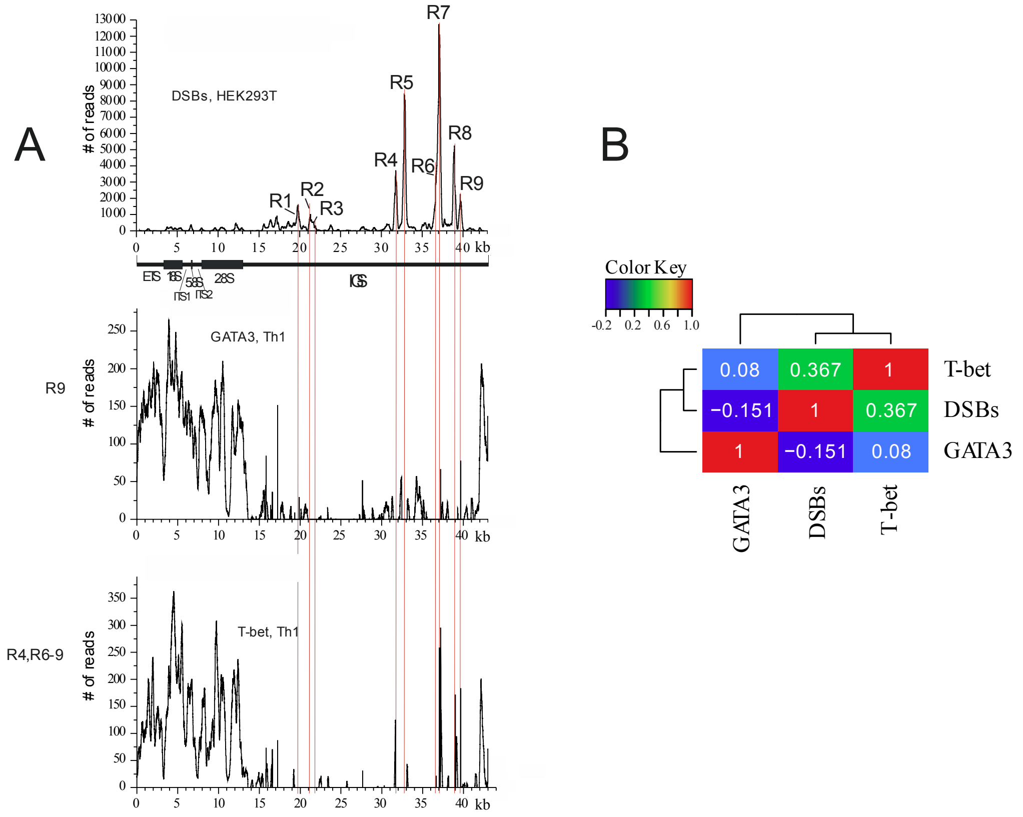

Fig. S5. The relationship between hot spots of DSBs, and GATA3, and T-bet binding sites inside rDNA units. (**A**) The profiles along a rDNA unit are shown. The thin red lines show the position of nine hot spots of DSBs (R1–R9). (**B**) Correlation heatmap of pairwise comparisons between median signals for DSBs in HEK293T cells, GATA3, and T-bet binding sites (both in Th-1 cells) inside the IGS are presented. Pearson correlation coefficients, r, are shown. There is a moderate positive correlation between DSBs and T-bet binding sites inside the IGS. T-bet binds within R4, and R6–R9 (similar to KAP1, see Fig. 4A and Table S1), while GATA3 binds only at R9.

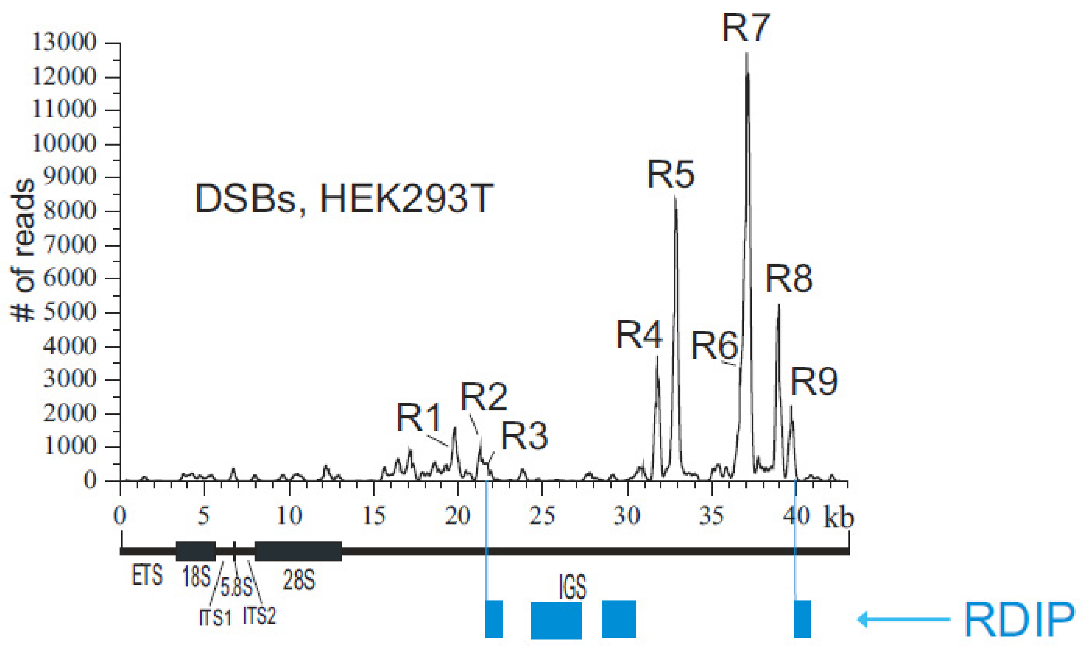

Fig. S6. **The relationships between hot spots of DSBs and RDIP (DNA:RNA immunoprecipitation) regions inside rDNA units**. The profile of DSBs along the rDNA unit is shown. The blue arrow indicates the position of RDIP regions detected inside rDNA units in IMR-90 cells (shown by blue bars) according to Nadel *et al*. (*27*).

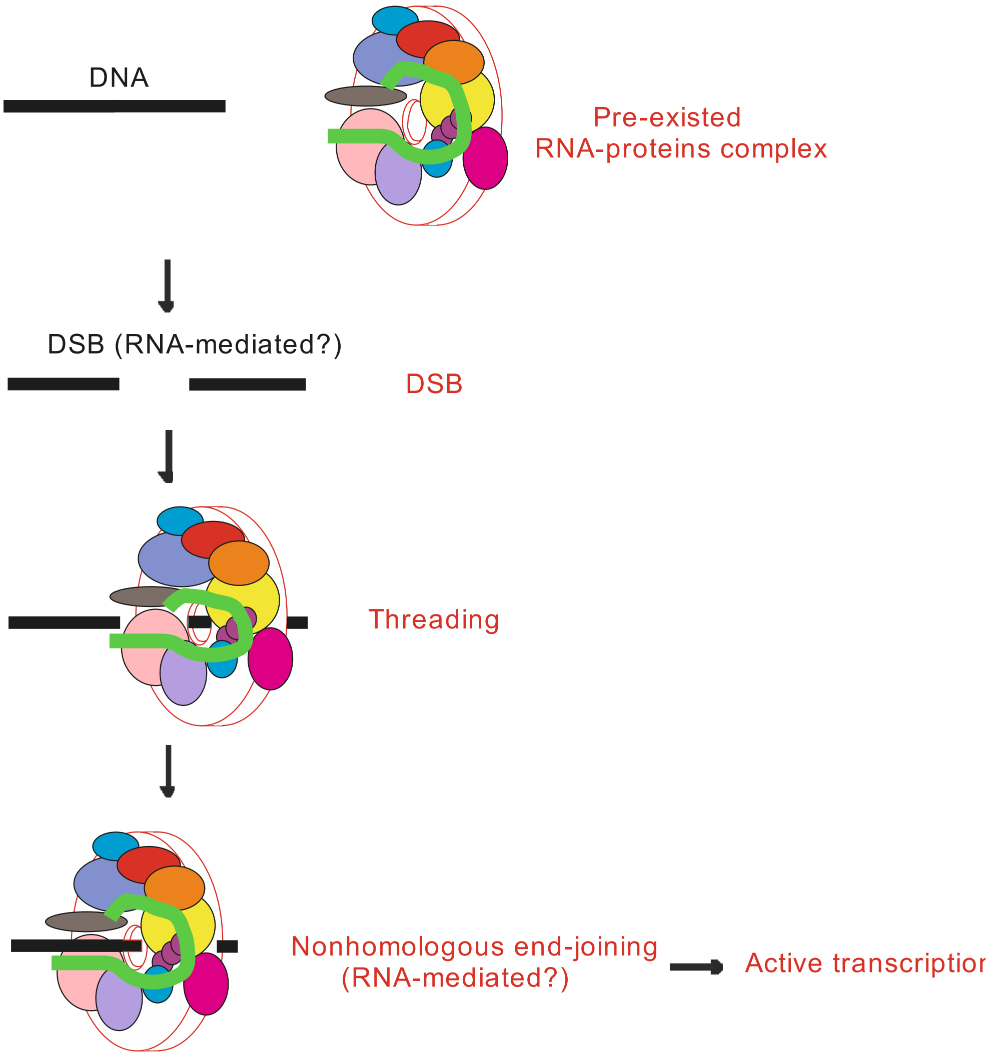

Fig. S7. A hypothetical model explaining the possible functional role of hot spots of DSBs inside rDNA units. The individual active rDNA units possess one particular hot spot of DSBs inside the IGS, which allows rapid loading of preexisting regulatory RNA-protein complexes. The assembly *in situ* of such complex structures could take much more time, while the suggested threading mechanism is rather fast. The physiological threading mechanism operates only for the rapid simultaneous activation of all rDNA units inside a particular cluster. The proposed model is based upon both the observations that hot spots of DSBs are only inside active rDNA clusters, and on the binding of different master regulators at sites possessing these spots.

Table S1. Binding of master regulators inside IGS regions containing hot spots of DSBs.

Factor/Region #	1	2	3	4	5	6	7	8	9
KAP1	+			+	+	+	+	+	+
TCF7L2		+		+	+	+	+		
GATA3									+
PARP1	+			+	+			+	
T-bet				+		+	+	+	+
CTCF	+	+	+	+	+	+	+	+	+
